# Influence of Interprofessional Care on Vancomycin Therapy Management in Hematology: A Retrospective Quality Improvement Analysis

**DOI:** 10.3390/antibiotics15101024

**Published:** 2026-10-15

**Authors:** Laura Sophie Schmidt, Henri Tong, Lilli Freund, Vincent-Christoph Klaus, Christoph Lübbert, Amrei von Braun, Henning Trawinski, Klaus H. Metzeler, Marco Herling, Uta Ceglarek, Thilo Bertsche, Yvonne Remane

**Affiliations:** 1 Hospital Pharmacy, Leipzig University Medical Center, 04103 Leipzig, Germany; laura.schmidt@medizin.uni-leipzig.de (L.S.S.); vincent-christoph.klaus@medizin.uni-leipzig.de (V.-C.K.); 2 Drug Safety Center, Medical Faculty, Leipzig University and Leipzig University Medical Center, 04103 Leipzig, Germany; thilo.bertsche@medizin.uni-leipzig.de; 3 Department of Clinical Pharmacy, Institute of Pharmacy, Medical Faculty, Leipzig University, 04103 Leipzig, Germany; 4 Department of Hematology, Cellular Therapy, Hemostaseology and Infectious Diseases, Leipzig University Medical Center, 04103 Leipzig, Germany; christoph.luebbert@medizin.uni-leipzig.de (C.L.); amrei.braun@medizin.uni-leipzig.de (A.v.B.); henning.trawinski@medizin.uni-leipzig.de (H.T.); klaus.metzeler@medizin.uni-leipzig.de (K.H.M.); marco.herling@medizin.uni-leipzig.de (M.H.); 5 Interdisciplinary Center for Infectious Diseases, Leipzig University Medical Center, 04103 Leipzig, Germany; 6 Comprehensive Cancer Center Central Germany, 04103 Leipzig, 07743 Jena, Germany; 7 Institute of Laboratory Medicine, Clinical Chemistry and Molecular Diagnostics, University Leipzig Medical Center, 04103 Leipzig, Germany; uta.ceglarek@medizin.uni-leipzig.de

**Keywords:** vancomycin, anti-infectives, trough levels, loading doses, administration, nephrotoxicity, hematology, consultation service

## Abstract

**Background:** Vancomycin therapy is challenging in hematology patients due to their complex therapies, frequent infections, variable renal function, and nephrotoxic co-medications, resulting in high pharmacokinetic variability. **Methods**: Vancomycin therapy was retrospectively analyzed over three consecutive periods using routine clinical data from a German tertiary academic hematology ward, focusing on recommended loading dose administration, therapeutic trough concentration attainment (15–20 mg/L), and a potential acute kidney injury (AKI): (I) baseline cohort (BC)—usual care; (IIa) infectious diseases support cohort (IDSC)—following collaboration with infectiologists; and (IIb) infectious diseases and pharmacy support cohort (IDPSC)—following additional clinical pharmacist involvement. **Results**: Overall, 155 patients receiving 164 vancomycin cycles were included. Recommended loading dose adherence increased from 3.3% (*n* = 3/90) in the BC to 32.6% (*n* = 15/46) in the IDSC and 53.6% (*n* = 15/28) in the IDPSC. The difference was statistically significant for both IDSC and IDPSC vs. BC (χ^2^ test, *p* < 0.001). Across cohorts, therapeutic trough concentrations were attained faster with the recommended than with an inadequate loading dose (median 4 vs. 8 days; log-rank test, *p* = 0.001). Subtherapeutic trough levels decreased (BC 78.6% vs. IDSC 65.5% vs. IDPSC 46.4%), while supratherapeutic levels increased (BC 7.6% vs. IDSC 10.8% vs. IDPSC 23.2%), accompanied by a higher AKI incidence (BC 20.0% vs. IDSC 17.4% vs. IDPSC 39.3%). The difference in AKI was statistically significant for IDPSC vs. BC (χ^2^ test, *p* = 0.038) and IDSC (χ^2^ test, *p* = 0.037). **Conclusions**: A recommended vancomycin loading dose within the interprofessional care cohort achieves therapeutic trough concentrations faster but requires close monitoring for nephrotoxicity.

## 1. Introduction

Patients with hematological malignancies represent a particularly vulnerable cohort with an increased risk of severe infections [1,2]. Rapid, targeted anti-infective treatment is essential, with careful consideration of individual factors, including organ function and concomitant medications to prevent life-threatening complications [3]. These patients are susceptible to severe Gram-positive infections, especially in the setting of neutropenia, such as those caused by central venous catheters and other medical devices [4,5]. Therefore, vancomycin, a glycopeptide antibiotic, is commonly used for the empirical and targeted treatment of suspected or confirmed infections, including those caused by methicillin-resistant *Staphylococcus aureus* (MRSA) [6,7]. The chosen dosages depend on various factors, including the patient’s body weight, renal function, and plasma concentration. Due to the narrow therapeutic range of vancomycin, therapeutic drug monitoring (TDM) is routinely performed to ensure therapeutic drug exposure while minimizing the risk of nephrotoxicity [6].

Current consensus guidelines recommend AUC-guided dosing, targeting an AUC_0–24_/MIC ratio of 400–600 for serious methicillin-resistant (MRSA) infections, as this approach provides a better balance between efficacy and nephrotoxicity than trough concentration-based monitoring [7]. Nevertheless, trough concentration monitoring remains an established component of routine clinical practice and is still widely used for vancomycin TDM. Previous guidelines recommended trough concentrations of 15–20 mg/L as a surrogate target range for adequate drug exposure [6,7]. Insufficient efficacy is expected below this therapeutic range (subtherapeutic levels), which may result in the development of resistance. Supratherapeutic levels are associated with an increased risk of adverse drug reactions. The most clinically significant adverse reaction is vancomycin-induced renal insufficiency, which may progress to acute kidney injury (AKI) [8,9]. The probability of developing AKI is increased by elevated plasma levels, prolonged therapy durations, and concomitant administration of other nephrotoxic medications [8,9,10,11].

According to the current German guideline on strategies to ensure rational antibiotic use in hospitals [12], interdisciplinary exchange forms the basis for continuous evaluation of indication, dosing, treatment duration, de-escalation strategies, and therapeutic outcomes. This highlights the importance of interprofessional collaboration among hematologists, clinical infectiologists physicians, and pharmacists [13,14,15]. Previous studies have focused on vancomycin-induced nephrotoxicity and its management through plasma concentration monitoring; however, data specifically addressing patients with hematological malignancies are lacking.

The aim of this analysis was to evaluate vancomycin therapy management across three consecutive interprofessional care periods, specifically focusing on the effect of the loading dose on the time to reach therapeutic plasma concentrations, the achievement of therapeutic trough levels, and the occurrence of AKI.

## 2. Results

### 2.1. Patient Characteristics

During the periods of observation, 198 patients were treated with vancomycin. Of these, 155 patients, who received 164 cycles of vancomycin, were analyzed. The patient cohort comprised 69 females (44.5%) and 86 males (55.5%). The median age was 62 years (Q25: 49; Q75: 69). A comprehensive overview of patients’ characteristics across the three observation periods is shown in Table 1.

### 2.2. Vancomycin Loading Dose

**Figure 1:**
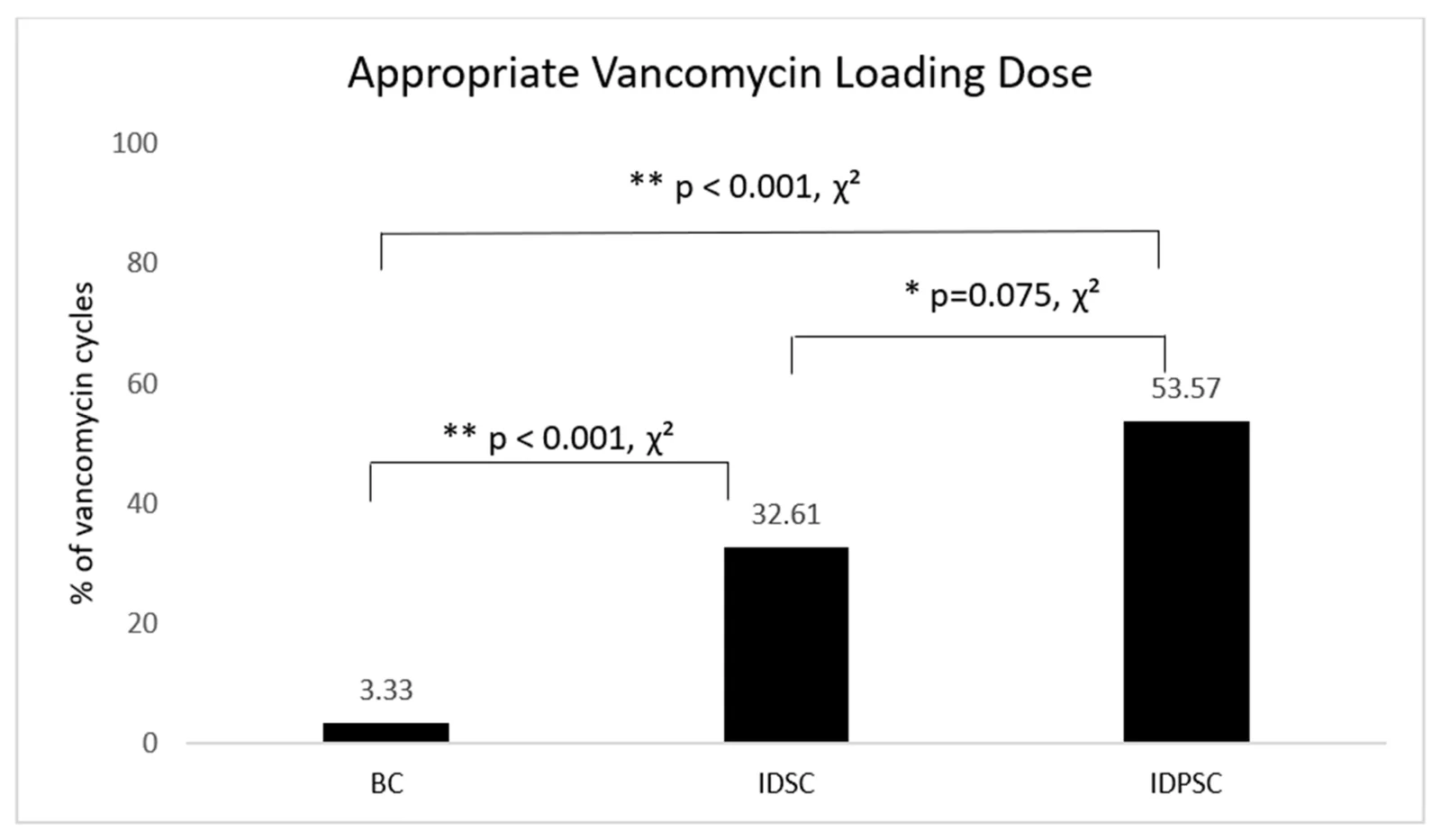
Percentage of vancomycin cycles with a vancomycin loading dose appropriate for the actual baseline kidney function in the BC (n = 3/90), IDSC (n = 15/46), IDPSC (n = 15/28). * No statistically significant difference in loading dose administration. ** Statistically significant differences in loading dose administration.

In the BC, 3.3% of vancomycin cycles started with the loading dose recommended for the actual baseline kidney function (*n* = 3/90). In the IDSC, this proportion increased to 32.6% (*n* = 15/46), while in the IDPSC, 53.6% (*n* = 15/28) of the cycles started with the recommended loading dose. Statistically significant differences in loading dose administration were found between BC and IDSC (χ^2^ test, *p* < 0.001) as well as between BC and IDPSC (χ^2^ test, *p* < 0.001). No statistically significant difference was found between IDSC and IDPSC (χ^2^ test, *p* = 0.075). A comparison is shown in Figure 1.

A total of 152 (out of 164) vancomycin cycles with documented loading doses and at least one measured trough level were included in the Kaplan–Meier analysis (Table 2). Twelve cycles were excluded due to missing follow-up trough level measurement data.

Of the 152 vancomycin cycles included in Kaplan–Meier analysis, the recommended loading dose for the actual baseline kidney function was administered in 30 vancomycin cycles (19.7%), while 122 vancomycin cycles (80.3%) received lower doses. Vancomycin cycles initiated with the recommended loading dose reached the therapeutic range within a median of 4 days [Q25 2; Q75 not estimable], compared to a significantly higher median of 8 days [Q25 4; Q75 15] for cycles without the recommended loading dose (*p* = 0.001 according to the log-rank test). The proportion of vancomycin cycles initiated with a recommended loading dose for the actual baseline kidney function increased progressively across the observation periods, from 3.3% (3/90) in the BC group to 32.6% (15/46) in the IDSC group and 53.6% (15/28) in the IDPSC. A Cochran–Armitage test for trend demonstrated a significant increasing trend (Z = 6.28, *p* < 0.001).

### 2.3. Vancomycin Trough Levels

A total of 836 vancomycin trough levels were analyzed. During the observation period, therapeutic vancomycin trough levels were not achieved in 61.6% of the vancomycin cycles in the BC, in 51.3% of the IDSC, and in 59.3% of the IDPSC, all within a single treatment cycle.

In the BC, 78.7% of vancomycin trough levels were below 15 mg/L, while 13.7% reached the therapeutic target range of 15–20 mg/L. In the IDSC, the proportion of subtherapeutic levels dropped to 65.5%, while 23.8% were within the target range. In the IDPSC, 46.4% had subtherapeutic levels, with 30.4% within the therapeutic range. Levels above 20 mg/L were most prevalent in the IDPSC at 23.2%, compared with 10.8% in the IDSC and 7.6% in the BC (Table 3).

While the proportion of subtherapeutic trough levels (<15 mg/L) decreased over time (BC 78.6% vs. IDSC 65.5% vs. IDPSC 46.4%), the proportion of supratherapeutic levels increased (BC 7.6% vs. IDSC 10.8% vs. IDPSC 23.2%), which was associated with a higher incidence of AKI (BC 20.0% vs. IDSC 17.4% vs. IDPSC 39.3%), which is described separately in Section 2.4.

A generalized estimating equation (GEE) model (GEE) accounting for repeated measurements within vancomycin treatment cycles demonstrated a significant overall effect of cohort on vancomycin trough concentrations (Wald-χ^2^ (2) = 11.84; *p* = 0.003). Estimated trough levels were 11.33 mg/L (95% CI 10.54–12.18) in the BC, 12.31 mg/L (95% CI 10.99–13.78) in the IDSC and 15.27 mg/L (95% CI 13.07–17.83) in the IDPSC. Bonferroni-adjusted pairwise comparisons showed significantly higher trough levels in the IDPSC than in the BC (mean difference, 3.94 mg/L; 95% CI 0.87–7.00; *p* = 0.006). No significant differences were observed between the BC and IDSC (mean difference, −0.98 mg/L; 95% CI −2.95 to 0.99; *p* = 0.703) or between the IDSC and IDPSC (mean difference, −2.96 mg/L; 95% CI −6.32 to 0.40; *p* = 0.105).

### 2.4. Assessment of Nephrotoxicity

Of 164 vancomycin cycles, 37 (22.6%) were associated with AKI. In the BC, the incidence of AKI was 20.0% (18/90). A subset of patients in the BC (5.5%, 5/90) were transferred to an intensive care unit (ICU), with 2 of these patients (40.0%) subsequently developing AKI. Within the IDSC, the incidence of AKI was 17.4% (8/46). In this cohort, 3 patients (6.5%) required intensive care, 2 of whom (66.7%) developed AKI. Within the IDPSC group, AKI was diagnosed in 39.3% (11/28). Here, four patients required intensive care treatment, of whom three (75.0%) developed AKI. No statistically significant difference regarding AKI was observed between the BC and IDSC groups. The incidence of AKI was significantly higher in the IDPSC group compared to the BC group (χ^2^ test, *p* = 0.038; 39.3% vs. 20.0%). Over the entire evaluation period, AKI occurred in 30.3% (10/33) of treatment cycles with a recommended loading dose for the actual baseline kidney function, compared with 20.6% (27/131) of those without the recommended loading dose. No statistically significant association was observed (χ^2^ test, *p* = 0.234). The prevalence of pre-existing renal insufficiency did not differ between the groups, and no differences were observed in the use of nephrotoxic co-medication.

## 3. Discussion

This retrospective analysis, conducted at a university tertiary care hospital and including 155 hematology patients receiving vancomycin therapy, demonstrated a significant increase in the use of recommended loading doses for the actual baseline kidney function following the introduction of collaborative care by infectious disease specialists and clinical pharmacists, compared with the pre-implementation baseline. Similar improvements in guideline adherence have been reported following the implementation of structured vancomycin treatment protocols, particularly within antibiotic stewardship (ABS) programs involving clinical pharmacists [17,18,19]. Nevertheless, Carland et al. reported that, despite the widespread availability of vancomycin guidelines, they are often not fully implemented, suggesting that additional interprofessional structures may be necessary to achieve a sustainable improvement in dosing practices [20].

When the recommended loading dose for the actual baseline kidney function was used, therapeutic trough concentrations of vancomycin were reached significantly faster than with underdosed initial regimens (median of 4 vs. 8 days). Our findings regarding the recommended loading dose are pharmacokinetically consistent with other analyses showing that the use of recommended loading doses significantly increases the likelihood of reaching therapeutic serum concentrations more rapidly [21,22].

Although target trough concentrations were reached earlier, the IDPSC showed a higher incidence of potentially vancomycin-associated AKI compared to both the BC and the IDSC. These findings indicate that earlier attainment of therapeutic trough concentrations does not necessarily reflect a safer or more appropriate vancomycin exposure. Trough level measurements within the target range of 15–20 mg/L represent only a single measurement point within the dosing interval; they do not allow for a reliable assessment of overall drug exposure [23]. The increased incidence of AKI could be related to the higher proportion of supratherapeutic vancomycin trough levels (>20 mg/L) in this group. Studies have described an association between elevated vancomycin concentrations and an increased risk of nephrotoxic events [9,11]. However, critically ill patients, particularly those with hematologic malignancies, have an increased risk of AKI even in the absence of vancomycin therapy [3,24].

No statistically significant association between the recommended loading dose for the actual baseline kidney function and the occurrence of AKI was found. The retrospective real-world design reflects routine clinical practice but also reveals gaps in the systematic documentation of established clinical risk factors. These gaps precluded a sufficiently robust multivariable analysis while identifying areas for improved documentation standardization and prospective evaluation.

Cycles receiving recommended loading doses for the actual baseline kidney function attain the predefined therapeutic trough concentrations earlier. Our study adds evidence from real-world data in patients with hematological malignancies and analyses loading-doses and subsequent trough target attainment within different models of interprofessional care. Ueda et al. found no significant differences in steady state trough concentrations despite an association with improved clinical response [21], whereas Yang et al. reported no significant improvement in early AUC target attainment with a 20 mg/kg loading dose [22]. The extent and intensity of interprofessional involvement were not systematically documented, limiting the assessment of individual interventions and their contribution to the observed outcomes. Thus, differences between the study periods, particularly in AKI rates, cannot be attributed solely to direct patient-specific interventions by infectious disease specialists or clinical pharmacists. Interprofessional collaboration may influence clinical practice through knowledge exchange, communication, education, and the dissemination of shared approaches to patient care [23].

Since trough concentrations alone do not reliably reflect overall vancomycin exposure [24], a switch from trough-level-based to AUC-based vancomycin therapy should be considered, particularly in patients at increased risk of nephrotoxicity or with substantial variability in renal function [8,25,26,27]. The 2020 consensus guideline recommends AUC-guided rather than trough-concentration-based monitoring targeting an AUC_0–24_/MIC ratio of 400–600 [7]. Model-informed precision dosing may support individualized vancomycin dosing in patients with hematological malignancies by incorporating patient-specific pharmacokinetic characteristics and covariates [28]. In addition, emerging machine learning approaches have been investigated for predicting vancomycin-associated AKI during loading dose administration [29] and for supporting individualized TDM [30]. However, the applicability of these models in patients remains to be established.

### Limitations

This analysis is based on routine data without prospective standardization. Consequently, the extent and intensity of interprofessional involvement were not systematically documented. Therefore, the contribution of individual intervention cannot be determined. As our retrospective analysis did not systematically capture established risk factors, such as sepsis, or treatment duration, their potential contribution to the observed AKI events cannot be excluded. Overall, the risk of AKI is multifactorial and is influenced by both patient- and treatment-related factors.

## 4. Materials and Methods

### 4.1. Setting and Study Design

The study was conducted at the Department of Hematology, Cellular Therapy, Hemostaseology and Infectious Diseases at the Leipzig University Hospital, a 1451-bed tertiary academic referral center in Germany. The department provides comprehensive inpatient and outpatient care for patients with hematologic malignancies and benign hematologic disorders, including stem cell transplantation and advanced cellular therapies. During the study period (2021–2025), a trough concentration of 15–20 mg/L was defined as the therapeutic target range according to the local vancomycin treatment protocol and used for the TDM analysis.

**Figure 2:**
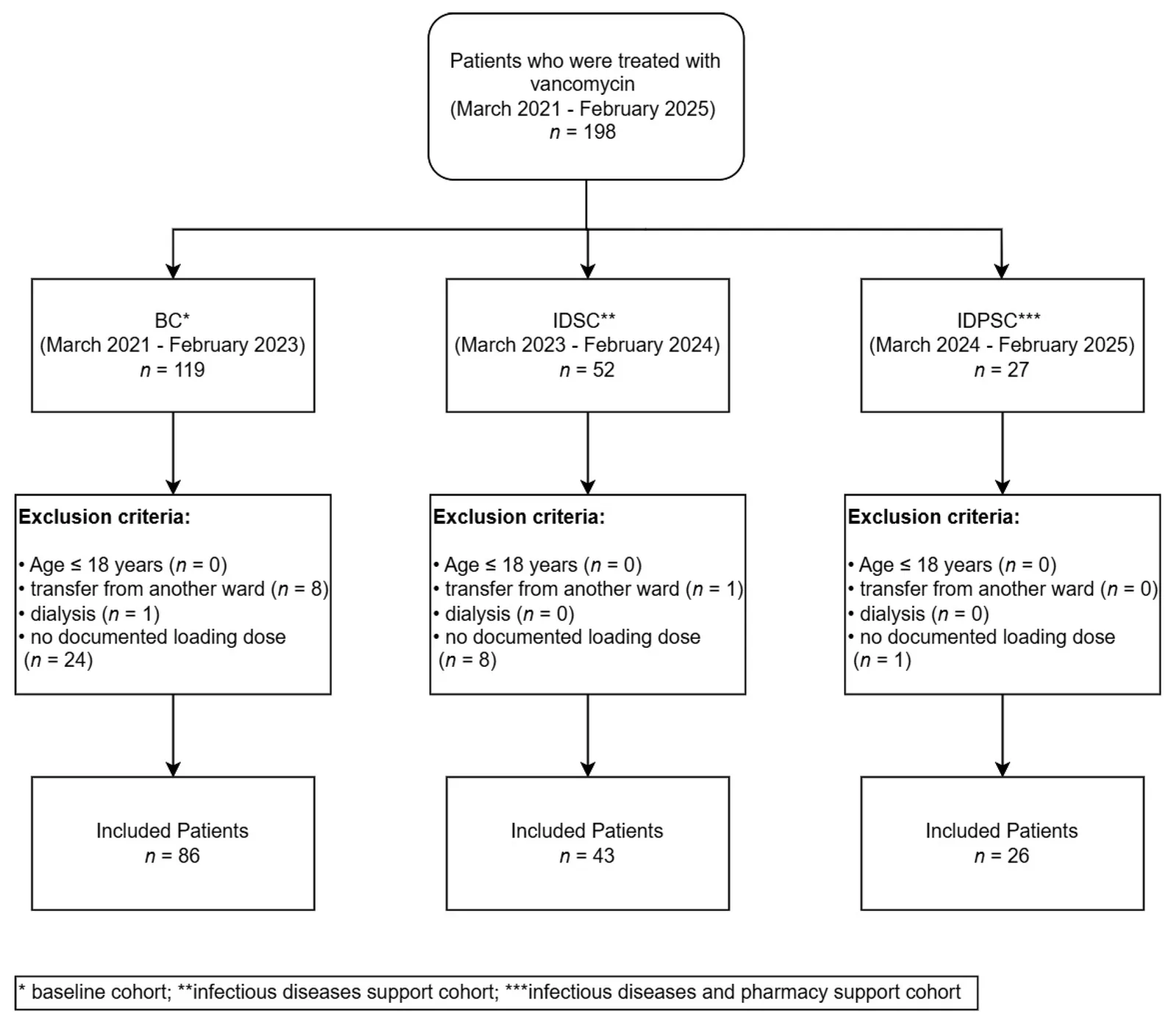
Patient selection across the three observation periods.

The study was reviewed and approved by the Ethics Committee of the Medical Faculty of Leipzig University, Germany, on 17 June 2025 (registration number 202/25-ek) and classified as a retrospective observational study evaluating routine clinical care, for which individual patient consent was not required. The study covered the period from 1 March 2021 to 28 February 2025 and was divided into three consecutive time periods: (I) baseline cohort (BC)—usual care (March 2021 to February 2023); (IIa) infectious diseases support cohort (IDSC)—integration of an infectious disease specialist into the care team (March 2023 to February 2024); and (IIb) infectious diseases and pharmacy support cohort (IDPSC)—implementation of a clinical pharmacist to support medication management (March 2024 to February 2025). The analysis included all adult patients (≥18 years) on a single ward who were not undergoing dialysis and who received intravenous vancomycin therapy with documented loading doses and at least one measured trough level (usually after at least 72 h) within the evaluation period (Figure 2). Following a loading dose, vancomycin was administered intravenously in multiple daily maintenance doses. To evaluate the vancomycin therapy management across three consecutive care models, the following factors were considered relevant: (i) the effect of the vancomycin loading dose on the time to reach therapeutic plasma concentrations, (ii) the influence of interprofessional care on achieving therapeutic trough levels, and (iii) the occurrence of AKI.

### 4.2. Data Collection and Evaluation

A systematic analysis of clinical data was performed to evaluate the initial vancomycin loading dose, trough concentrations, and occurrence of AKI. Recommended loading doses were defined as 25–30 mg/kg of body weight (maximum, 3 g) [7]. According to the local vancomycin treatment protocol, patients with renal impairment, defined as an estimated glomerular filtration rate (eGFR) of <90 mL/min, should receive a reduced dose of 20–25 mg/kg of body weight [31]. Documented body weight and dosing data were reviewed to determine adherence to these recommendations. Therapeutic trough concentrations were defined as 15–20 mg/L [7]. The assessment of nephrotoxicity was conducted in accordance with the Kidney Disease Improving Global Outcomes (KDIGO) criteria, with AKI defined by an increase in serum creatinine of ≥26.5 µmol/L (≥0.3 mg/dL) within 48 h, a rise to ≥1.5 times baseline within seven days, or urine output of <0.5 mL/kg/h over six hours [16].

Patients receiving vancomycin therapy at the time of inter-unit transfer were excluded because only electronic medical record data from the hematology unit were available for analysis. Vancomycin plasma concentrations obtained more than 24 h after the last administered dose were excluded from the analysis. Patients who did not reach therapeutic trough concentrations were treated as right-censored observations.

### 4.3. Statistical Analysis

Statistical analysis was conducted using IBM SPSS Statistics for Windows, version 29 (IBM Corporation, Armonk, NY, USA). Continuous variables are presented as median [25th (Q25) and 75th (Q75) percentiles]. Treatment cycles were considered the primary unit of analysis and were assumed to be independent observations, as repeated cycles within the same patient were separated by substantial time intervals. No formal adjustment for within-patient correlation was performed. Differences in the proportion of cycles receiving recommended loading doses for the actual baseline kidney function, developing AKI, or attaining therapeutic trough concentrations were analyzed using Pearson’s chi-square test (χ^2^ test). For comparisons of proportions across the three sequential care models (BC, IDSC, and IDPSC), the Cochran–Armitage test for trend (Z) was used to assess whether the respective predefined criterion changed systematically across the analyzed cohorts.

A total of 836 vancomycin trough concentrations were analyzed using the Mann–Whitney U test for comparisons between two groups, whereas the GEE model was used for comparison between multiple groups to account for the correlation between repeated measurements within the same treatment cycle. A gamma distribution with a log link and an exchangeable working correlation structure was used. Robust standard errors were applied. Pairwise comparisons were Bonferroni-adjusted, and *p* < 0.05 was considered statistically significant. Time to first attainment of a vancomycin trough concentration within the predefined targeted therapeutic range was evaluated using Kaplan–Meier analysis. Follow-up started with the first documented trough concentration of a treatment cycle and ended at first attainment of the target range or, if target range was not attained, at the least available concentration during the cycle.

Differences were assessed with the log-rank test. A two-sided *p*-value < 0.05 was considered statistically significant.

## Figures and Tables

**Table 1:** Overview of patient characteristics across the three observation periods.

		BC *		IDSC **		IDPSC ***	
		Median	Q25; Q75	Median	Q25; Q75	Median	Q25; Q75
Age [y]		62	49; 68	60	48; 68	65	58; 70
Gender	Female	39 (45.3%)		18 (41.9%)		12 (46.2%)	
	Male	47 (54.7%)		25 (58.1%)		14 (53.8%)	
Diagnosis	Myeloid neoplasms	63 (73.2%)		26 (60.5%)		13 (50.0%)	
	Lymphoid neoplasms	17 (19.8%)		11 (25.6%)		8 (30.8%)	
	Others	6 (7.0%)		6 (13.9%)		5 (19.2%)	
^1^ Basal renal function according to KDIGO ****	G1	50 (55.6%)		25 (54.3%)		15 (53.6%)	
	G2	25 (27.8%)		16 (34.8%)		13 (46.4%)	
	G3	13 (14.4%)		5 (10.9%)		0 (0.0%)	
	G4	2 (2.2%)		0 (0.0%)		0 (0.0%)	
	G5	0 (0.0%)		0 (0.0%)		0 (0.0%)	
^1^ Antibiotic Therapy	Empiric	66 (73.3%)		33 (71.7%)		22 (78.6%)	
	Targeted	24 (26.7%)		13 (28.3%)		6 (21.4%)	
Causative Pathogens for Targeted Therapies		*S. epidermidis* (41.7%) *E. faecium* (16.7%) *S. hominis* (12.5%) *S. aureus* (12.5%) *S. haemolyticus* (8.3%) *S. capitis* (4.2%) Methicillin-resistant *S. aureus* (4.2%)		*S. epidermidis* (38.5%) *E. faecium* (23.1%) *S. hominis* (7.7%) *C. striatum* (7.7%) *S. haemolyticus* (7.7%) *E. avium* (7.7%) Methicillin-resistant *S. aureus* (7.7%)		*S. epidermidis* (50.0%) *E. faecium* (50.0%)	
MIC of Vancomycin for Targeted Therapies	≤0.5 µg/mL	8 (33.3%)		4 (30.8%)		0 (0.0%)	
	≤1 µg/mL	12 (50.0%)		8 (61.5%)		6 (100.0%)	
	≤2 µg/mL	4 (16.7%)		1 (7.7%)		0 (0.0%)	
^2^ Leukocytes *	<1.5 × 10^9^/L	63 (70.0%)		27 (58.7%)		19 (67.9%)	
	>1.5 × 10^9^/L	27 (30.0%)		19 (41.3%)		9 (32.1%)	

* Baseline cohort; ** infectious diseases support cohort; *** infectious diseases and pharmacy support cohort. **** KDIGO GFR categories: G1, ≥90 mL/min/1.73 m^2^ (normal or high); G2, 60–89 (mildly decreased); G3, 30–59 (moderately decreased); G4, 15–29 (severely decreased); G5, <15 (kidney failure) [16]. ^1^ Based on vancomycin cycles. ^2^ At the time of vancomycin administration.

**Table 2:** Overview of vancomycin cycles reaching the target vancomycin trough concentrations in the Kaplan–Meier analysis.

Outcome	BC (*n* = 86)	IDSC (*n* = 39)	IDPSC (*n* = 27)
Reached Target	33	19	11
Not Reached, Censored	53	20	16
Missing, Excluded	4	7	1

**Table 3:** Trough Level Concentrations by Observation Period.

**Trough Level**	**BC (*n* = 488)**	**IDSC (*n* = 223)**	**IDPSC (*n* = 125)**
>20 mg/L (supratherapeutic)	37 (7.6%)	24 (10.8%)	29 (23.2%)
15–20 mg/L (therapeutic)	67 (13.7%)	53 (23.8%)	38 (30.4%)
<15 mg/L (subtherapeutic)	384 (78.7%)	146 (65.5%)	58 (46.4%)

## Data Availability

Anonymized data will be available upon request. Contact yvonne.remane@medizin.uni-leipzig.de.
